# RBE-weighted dose conversions for patients with recurrent nasopharyngeal carcinoma receiving carbon-ion radiotherapy from the local effect model to the microdosimetric kinetic model

**DOI:** 10.1186/s13014-020-01723-z

**Published:** 2020-12-10

**Authors:** Liwen Zhang, Weiwei Wang, Jiyi Hu, Jiade Lu, Lin Kong

**Affiliations:** 1 Department of Medical Physics, Shanghai Proton and Heavy Ion Center, Fudan University Cancer Hospital, Shanghai, 201321 China; 2 Shanghai Engineering Research Center of Proton and Heavy Ion Radiation Therapy, Shanghai, China; 3 Department of Medical Physics, Shanghai Proton and Heavy Ion Center, Shanghai, 201321 China; 4 Department of Radiation Oncology, Shanghai Proton and Heavy Ion Center, Shanghai, 201321 China; 5 Department of Radiation Oncology, Shanghai Proton and Heavy Ion Center, Fudan University Cancer Hospital, Kangxin Road No. 4365, Shanghai, 201321 China

**Keywords:** Carbon ion radiotherapy, LEM I, MKM, RBE-weighted doses

## Abstract

**Background:**

We sought to establish a conversion curve to convert the RBE-weighted doses calculated by local effect model I (LEM) (LEM RBE-weighted doses) in patients with locally recurrent nasopharyngeal carcinoma (rNPC) to the RBE-weighted doses calculated by microdosimetric kinetic model (MKM) (MKM RBE-weighted doses). We also converted the LEM dose constraints (RBE-weighted dose constraints in LEM plans) for the brain stem, spinal cord, and optic nerve based on this curve.

**Methods:**

Data from 20 patients with rNPC receiving carbon-ion radiotherapy (CIRT) in our hospital were collected. LEM in Raystation (V8A, Raystation, Sweden) was used to generate treatment plans. The clinical target volume CTV1 (GTV + 5 mm) was given 3 Gy (RBE) per fraction. Ninety-nine percent of target volumes should be covered by 95% of the prescriptions; the maximum doses of the brainstem and spinal cord were < 45 Gy (RBE) and < 30 Gy (RBE), respectively. The doses covering 20% volumes of optical nerves/chiasms D20 were < 30 Gy (RBE). Then physical doses of the LEM plans were recalculated by using MKM in Raystation to generate MKM plans. A series of conversion factors (i.e., the ratio of LEM RBE-weighted dose to MKM RBE-weighted dose) was then obtained by using an isovolumetric dose method. The LEM plan prescriptions (LEM prescription) and dose constraints of the organs at risk (OARs) (OAR constraints) were converted to the corresponding MKM prescriptions and dose constraints using this conversion curve.

**Results:**

For the CTV1 fractional RBE-weighted dose prescription of 3.00 Gy (RBE) and CTV2 of 2.70 Gy (RBE) in LEM plans, the conversion factors (LEM RBE-weighted dose/MKM RBE-weighted dose) were 1.37 (CI 95% 1.35–1.39) and 1.46 (1.41–1.51), respectively. The average conversion factors from 1.37 (CI 95% 1.33–1.41) to 3.09 (2.94–3.24) corresponded to the LEM fractionated doses from 2.86 Gy (RBE) to 0.24 Gy (RBE), including the doses constraining upon OARs. LEM RBE-weighted doses of 30 Gy (RBE) and 45 Gy (RBE) in 21 fractions were converted to MKM RBE-weighted doses of 16.64 Gy (RBE) and 30.72 Gy (RBE) in 16 fractions.

**Conclusions:**

This conversion curve could be used to convert LEM RBE-weighted doses to MKM RBE-weighted doses for patients with rNPC receiving CIRT, providing dose references for re-irradiation therapy.

## Background

Previous studies [1–3] have shown that particle radiotherapy, such as carbon ions, has significant dosimetric advantages over photon radiation. Our center—The Shanghai Proton and Heavy Ion Center (SPHIC)—has been using carbon ions to treat patients with locally recurrent nasopharyngeal carcinoma (rNPC) since May 2015. A follow-up of 75 patients to 2017 showed a survival rate of 82.2% without local progression [4]. These outcomes are significantly better than those of patients receiving photon re-irradiation. The National Institute of Radiobiological Sciences (NIRS, Japan) started carbon ion radiotherapy in 1994 [5, 6]. Up until July 2017, NIRS has treated up 11,580 patients [7], whose experiences with dose prescription and dose constraints of the organs at risk (OARs) (OAR constraints) deserve communication. However, the biophysical models used in these two carbon ion centers are different. NIRS uses the modified Microdosimetric Kinetic Model (mMKM) [8–10]. The mMKM we reference in this paper is hereafter referred to as MKM and is used for treatment planning. Our institute uses the local effect model I (LEM) [11, 12] for treatment planning. MKM assumes the RBE of carbon-ion peaks when the dose-averaged linear energy transfer (LETd) is 100–150 keV/um [8] and ignores the RBE dependence on doses [10]. However, LEM assumes that RBE relies on the spatial dose distributions of carbon ion and depends on the delivered doses [11]. Therefore, the two systems calculate different RBE-weighted doses, even based on the same physical doses.

To refer to the clinical experience of NIRS, the LEM group used the new LEM prescription converted from the corresponding MKM prescription while still using the original unconverted MKM OAR constraints [13–15]. However, clinical evidence collected over years of applications showed that this way made the planning too difficult, and the OAR constraints were too conservative. Adaptation of the OAR constraints was absolutely necessary. So far, only LEM dose constraints of the optical nerve [16] and rectum [17, 18] have been reformulated; no studies have been carried for recurrent tumors (e.g., rNPC). Since the OARs of patients with rNPC (e.g., optical nerve, spinal cord, and brain stem) have already exposed to a sufficient amount of radiation, stricter OAR constraints should be discussed.

This work not only focused on targets in patients with rNPC but expanded to OAR stand. We also compared our experience with RBE-weighted dose constraints for patients with rNPC with those in NIRS [19] and the current dose constraints on optic nerve in the National Center of Oncological Hadrontherapy, Italy (CNAO) [16] to validate the safety of re-radiation and establish a reference for adjusting the prescription dose for patients with head and neck cancer.

## Methods

### Patient selection and planning

We randomly selected 20 local patients with rNPC who underwent CIRT at our hospital from June 2016 to December 2017.

Target definition [4]: The gross tumor volume (GTV) includes visible tumor lesions on CT, PET-CT, and MRI. The clinical target volumes (CTV1) of both the GTV of the primary site and neck were designed to include 5 mm beyond the GTV for microscopic extension (limited to as little as 1 mm near OAR), and a variable margin for occult tumor spread. CTV2 includes CTV1 and subclinical lesions that may be invaded by the tumor. The planning target volume (PTV) is based on a CTV expansion of 6 mm in the direction lateral to the beams and 3 mm in other directions, which is calculated based on the range uncertainty [20] and allows for setup variability and uncertainty about dose distribution. Optimizing PTV can help meet target dose requirements.

Prescription: Information obtained from selected patients is listed in Table 1. Their dose per fraction was the same 3.00 Gy (RBE) of CTV1 and 2.70 Gy (RBE) of CTV2. Since the dose conversion is only related to the dose per fractions [13], the total fractionations of all patients were rescaled to 21 fractions in the new treatment plans.

**Table 1:** The irradiation parameters (stage and LEM dose prescription) for all the patients

Patient	TNM Stage	Clinical stage	LEM prescription/Gy (RBE)	Fraction
P01-P03	T4N0M0	IV	63	21
P04-P06	T3N1M0	III	63	21
P07	T3N0M0	III	63	21
P08	T2N0M0	II	63	21
P09	T1N0M0	I	63	21
P10-P12	T4N0M0	IV	60	20
P13	T3N0M0	III	60	20
P14	T2N0M0	II	60	20
P15	T1N0M0	I	60	20
P16	T4N0M0	IV	57	19
P17	T3N0M0	III	57	19
P18	T2N1M0	II	57	19
P19	T3N2M0	III	54	18
P20	T3N0M0	III	54	18

**Fig. 1:**
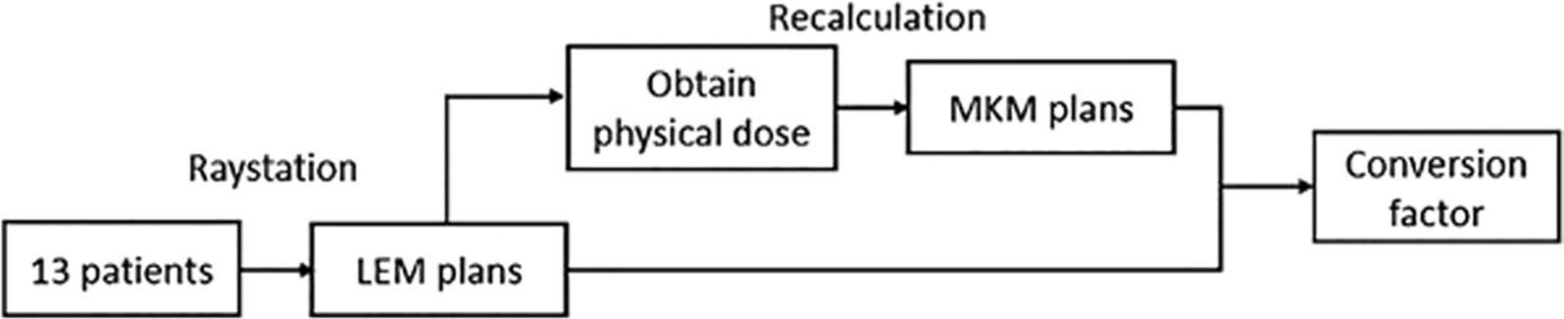
The flowchart of treatment planning and dose conversion

**Fig. 2:**
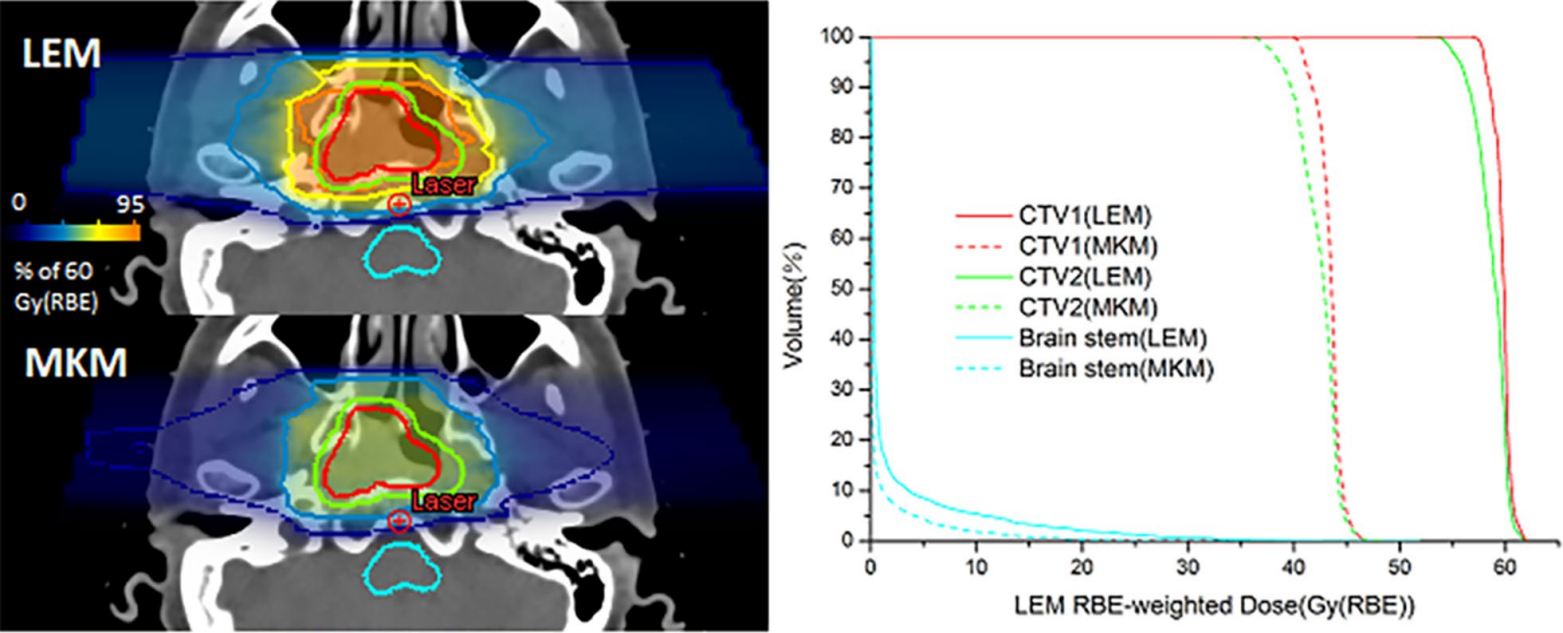
Left side: transversal view of one patient LEM and MKM RBE-weighted dose distributions with CTV1 (red), CTV2 (green) and brain stem (cyan) contours. The orange coverage is 95% of the prescribed dose. Right side: corresponding dose volume histograms (DVHs) of CTV1 (red), CTV2 (green) and brain stem (cyan) in LEM plans (solid line) and MKM plans (dash line)

This study was performed using the Raystation (V8A, Raysearch, Sweden) treatment planning system, which incorporates both MKM and LEM. The RBE-weighted doses calculated by LEM and MKM are hereafter referred to as the LEM RBE-weighted dose and MKM RBE-weighted dose. Plans of selected patients, based on LEM, were first generated as LEM plans. The plan pass criteria [4] for the LEM plan are listed in the second column in Table 2. Next, MKM [10] was used to recalculate the physical doses obtained from the optimization of the LEM plan and created corresponding MKM plans to obtain the MKM RBE-weighted dose distributions. Figure 1 shows the flowchart of treatment planning and dose conversion for one patient. Figure 2 shows an example of treatment planning for one patient.

**Table 2:** OAR constraints under MKM obtained from the conversion curve

OAR constraints/Gy (RBE)	LEM constraint (21 fractions)	LEM constraint (16 fractions)	MKM constraint (16 fractions)	70% NIRS constraints	Conversion factor
Brain stem Dmax	45.00	43.68	30.72 (30.71–30.73)	28.00	1.42 (1.40–1.44)
Spinal cord Dmax	30.00	29.28	16.64 (16.63–16.65)	21.00	1.76 (1.74–1.78)
Optic nerve D20	30.00	29.28	16.64 (16.63–16.65)	19.60	1.76 (1.74–1.78)

### Isovolumetric dose method

Wang [18] has analyzed the feasibility of RBE-weighted dose conversion from MKM to LEM. Hence, if the physical dose and fragment spectrum are exactly the same, the LEM isodose can be transformed into a defined MKM isodose. Also, under the clinical treatment plan, they have the same biological equivalent dose-volume, which is a more efficient tool to establish the conversion relationship.

### Conversion curve

Previous scholars [15] defined the conversion factor as the ratio of the LEM RBE-weighted dose to the MKM RBE-weighted dose. For the dose conversion inside CTV, we directly focused on the dose in the target volume of CTV1 and CTV2 in the LEM and MKM plan of each patient.

For the dose conversion outside CTV, we first defined the dose area of interest outside the CTV as the CTV 20 mm extension (exclude CTV), which includes all the OAR adjacent to the CTV. Then, 56 isodose curves of 60.00 Gy (RBE) to 5.00 Gy (RBE) were selected in the LEM plans of 13 patients, whose fractional doses ranged from 2.86 Gy (RBE) to 0.24 Gy (RBE). The volume of each isodose line was obtained, and then the corresponding MKM RBE-weighted dose of the same volume was found in the MKM plan. A series of conversion factors were obtained according to the definition.

For patient 01, the RBE-weighted isodoses for the LEM plan were: 2.86 Gy (RBE), 2.81 Gy (RBE), 2.76 Gy (RBE), etc. The corresponding volumes of these isodoses are 8.25 cubic centimeters (cc), 12.12 cc, 17.27 cc. For the same volume, the RBE-weighted isodoses in MKM plan were 2.05 Gy (RBE), 2.00 Gy (RBE), and 1.95 Gy (RBE), whose conversion factors in such LEM RBE-weighted doses were 1.39, 1.41, and 1.42.

### The conversion of the OAR constraints

Most patients with head and neck tumors in NIRS received 16 fractions of radiation, while the methods of this study used 21 fractions, as described above. Since the total dose in multi-fraction irradiations depends more on the size of dose-per-fraction for late, rather than for early, damage to normal tissues [21], the Linear-quadratic (LQ) model should be first used to convert the dose limits in 21 fractions of the LEM plan to the dose limits in 16 fractions. Under the same fractionations, a single MKM RBE-weighted dose can be obtained by using the conversion curve in this study. Finally, we multiplied the single dose of MKM by 16 to obtain the total corresponding MKM RBE-weighted dose for the 16 fractions.

## Results

### The conversion curve inside CTV

Table 3 now presents the results of conversion factor inside CTV for 20 patients. P20 did not have CTV2 since the plan was a boost treatment after irradiation with proton. Based on the 20 cases, the average conversion factors were 1.37 (CI 95% 1.35–1.39) and 1.46 (1.41–1.51) for LEM RBE-weighted dose 3.00 Gy (RBE) of CTV1 and 2.70 Gy (RBE) of CTV2. The corresponding MKM RBE-weighted doses were 2.18 (2.15–2.21) Gy (RBE) and 1.85 (1.79–1.91) Gy (RBE). According to the fitting results of the Fossati curve [13], the dose percentage differences ((Conversion factor-1) × 100%) of the above MKM RBE-weighted doses were 38.00% and 47.20%. However, the same dose percentage differences based on our study were 37.27% and 45.90%, indicating an approximately 0.73–1.3% deviation.

**Table 3:** CTV prescription (3 Gy (RBE) of CTV1 and 2.7 Gy (RBE) of CTV2) in LEM plans (LEM prescription) and corresponding conversion factor of 20 patients

LEM prescription	Conversion factor									
	P01–P20									
3.0 Gy (RBE)	1.35	1.38	1.33	1.37	1.38	1.40	1.36	1.34	1.38	1.39
	1.39	1.37	1.39	1.36	1.39	1.36	1.38	1.39	1.38	1.35
2.7 Gy (RBE)	1.67	1.45	1.42	1.47	1.45	1.45	1.45	1.44	1.44	1.45
	1.47	1.46	1.47	1.44	1.46	1.41	1.44	1.45	1.46	/

### The conversion curve for RBE-weighted isodose volumes outside CTV

**Fig. 3:**
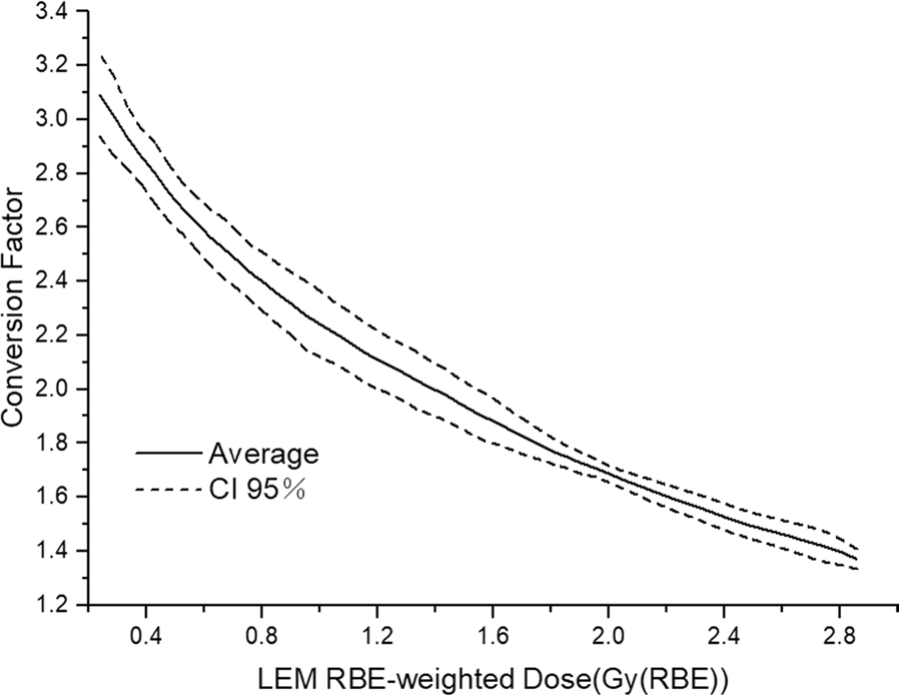
Conversion curve from LEM to MKM for dose region outside CTV in 20 patients with rNPC (black solid line represents the average value and dashed lines the 95% confidence interval (CI))

As the conversion factors were obtained by the isovolumetric dose method, Fig. 3 shows the conversion curve outside the CTVs based on our results. The horizontal axis is the fractional dose (0.24–2.86 Gy (RBE)/fraction), and the vertical axis is the conversion factor corresponding to LEM to MKM [1.37 (CI 95% 1.33–1.41) to 3.09 (2.94–3.24)].

### Example of isodose curve conversion

**Fig. 4:**
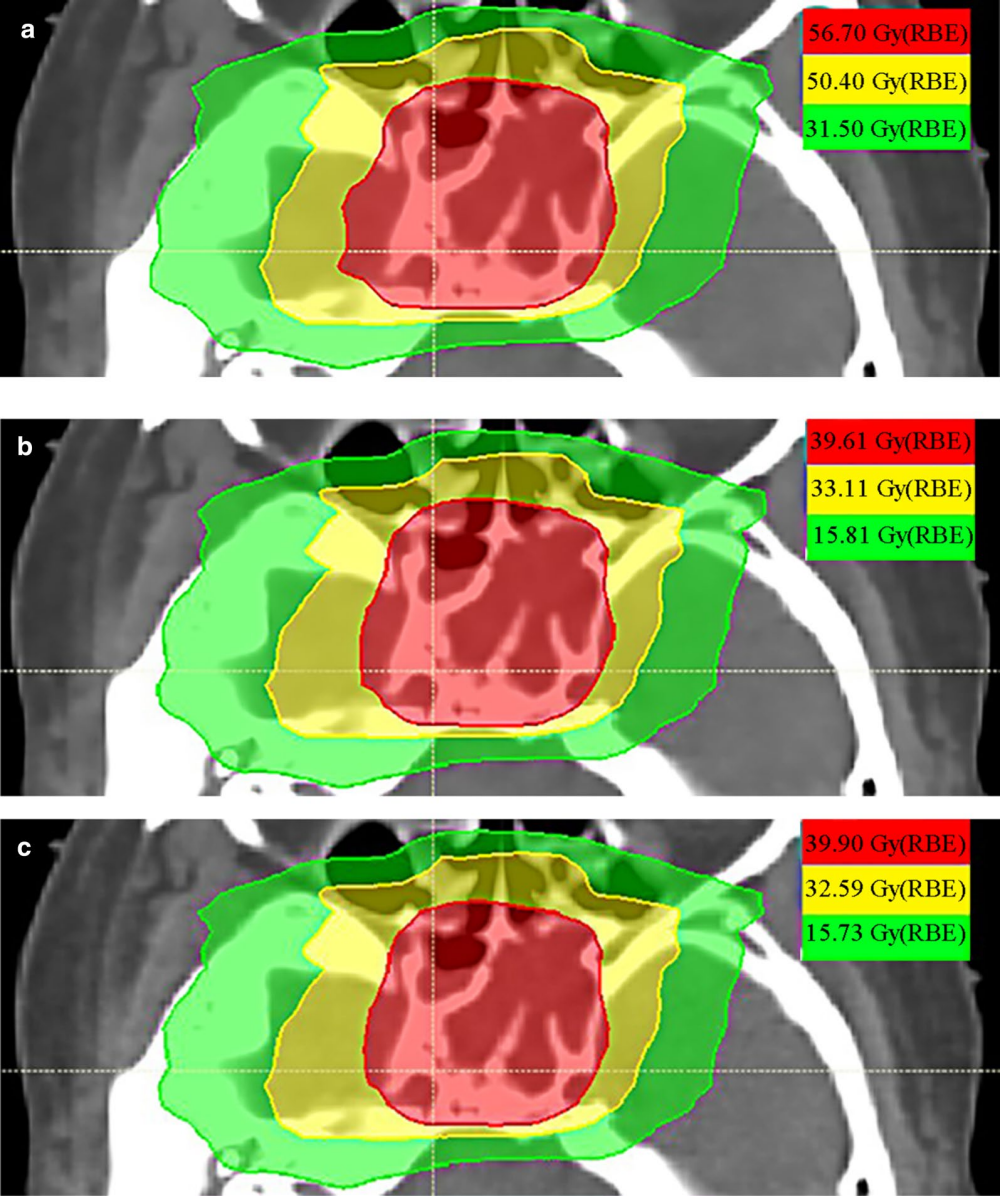
The RBE-weighted dose distribution (three dose lines) from a patient with rNPC in LEM plan (**a**), the dose distribution in recalculated MKM plan (**b**) and the dose distribution using the conversion curve in MKM plan (**c**); **b** and **c** are nearly identical

Figure 4 shows the dose distributions of the LEM plan and corresponding MKM plan from a patient with rNPC. Three low, medium, and high dose lines of 31.50 Gy (RBE) (Volume covered by 50% of the prescription dose, V50), 50.40 Gy (RBE) (V80), and 56.70 Gy (RBE) (V90) are displayed under LEM plan as shown in Fig. 4a, whose volumes are 3.33 cubic centimeters (cc), 2.00 cc, and 0.24 cc, respectively. Figure 4b shows the corresponding isodose lines of 15.81 Gy (RBE), 33.11 Gy (RBE), 39.61 Gy (RBE) of the MKM RBE-weighted dose in the same volume. Using the conversion curve to obtain the conversion factor, we could deduce that the dose distributions of 15.73 Gy (RBE), 32.59 Gy (RBE) and 39.90 Gy (RBE) in the recalculated MKM plan (21 fractions) were the same as that of the three-dose lines in the LEM plan (as shown in Fig. 4c). Considering the error of the conversion factor curve, the image is only slightly different (Fig. 4b, c).

### The conversion of the OAR constraints

The OAR limits of the carbon ion plan for patients with rNPC [4] in our hospital are shown in the second column of Table 1. Since our hospital uses 21 fractions of radiotherapy for rNPC and NIRS uses 16 fractions of radiotherapy for head and neck tumors, the LQ model was used to convert the dose of 21 fractions of LEM plan to 16 fractions [21], as shown in the third column. Then the corresponding dose limit of the MKM plan was converted according to the average curve of Fig. 3. The limits to OARs for the first radiotherapy (16 fractions) of head and neck tumors in NIRS are maximum brainstem dose of 40.00 Gy (RBE) (single dose of MKM 2.50 Gy (RBE)) [19], maximum spinal cord dose of 30.00 Gy (RBE) (MKM single dose 1.88 Gy (RBE)) [19], and dose covering 20% volumes of optical nerves/chiasms D20 < 28.00 Gy (RBE) (MKM single dose 1.75 Gy (RBE)) [16, 22]. Considering re-irradiation therapy, all the OAR dose limits in our hospital are compared with 70% of the limits mentioned above for NIRS [23]. Aside from the fact that the brainstem was slightly higher than 70% of the NIRS limit, the rest were within limits and belong to the “safe range.”

## Discussion

The conversion factor in targets have a possible deviation > 1%. This is considered reasonable since the patient’s target area involves a combination of various factors (target size, dose, depth of target area, beam configuration, etc.). The conversion curve outside CTV is also consistent with the previous findings of the other scholars [13, 15]: as the prescription dose increases, the difference between the LEM RBE-weighted dose and the MKM RBE-weighted dose gradually decreases. That is, the conversion factors decrease with increases in the fractional dose and eventually approach 1.00. These results all indicate that the physical dose, as well as the RBE-weighted dose of OARs, will be lower than those in MKM plans if the same prescription is used. Thus, examination of the conversion relationship between the two models are necessary to help unify the experience across different cancer centers. We further extended the dose range for conversion to OAR to account for a broader exchange of biological doses. However, the LEM RBE-weighted doses of some patients could not find the corresponding MKM RBE-weighted dose value in our study since the calculated LEM RBE-weighted doses are always greater than the corresponding MKM RBE-weighted dose within dose range based on the same physical dose. So the target's fractional dose in this article is the maximum prescribed dose of 3 Gy (RBE), and the maximum fractional dose outside CTV is 95% of the maximum prescribed dose, which is 2.86 Gy (RBE).

This study was performed on patients with rNPC. Compared with patients with primary tumors, patients with rNPC had a significantly reduced tolerated doses to the organ. The conversion study was mainly based on the physical parameters of the carbon-ion beam (i.e., tumor size and location, beam setup, etc.). The radio-resistance of rNPC was not considered in the RBE calculation and conversion. However, since rNPC has OARs like the brain stem, spinal cord, and the optic nerve for head and neck cancers, the converted results should be applicable to other head and neck tumors with similar locations. However, different cancers carry slightly different conversion factors (ongoing research). Although NIRS has not reported any experience with treating rNPC, data collected over the long-term indicate normal tissue damage after the initial treatment of head and neck tumors. Based on Nieder’s study [23], our clinical trials finally decided to start with 70% of their MKM corresponding dose constraints as the safe criterion. We then validated the safety of re-radiation with rNPC in LEM plans so as to set an initial reference for rNPC. What’s more, Jon et al. [16] in CNAO found the relationship between D_NIRS_ and D_LEM_ for D_1%_ and D_20%_. They evaluated cumulative dose-volume histograms (DVHs) of all optic nerves to determine a less conservative constraint for a 16 fraction CIRT treatment by analyzing institutional toxicity and by relating the results to the constraints validated by NIRS. In Jon et al.’s research, D_20%_ is 37 Gy (RBE), a little higher than our 29.28 Gy (RBE) (16 fractions), which is compliant considering re-irradiation in SPHIC.

During follow-up—up to December 2018—none of patients in this study had serious neurotoxic side effects. This further verified the safety of the dose constraints for important nerve endangering organs (brainstem, spinal cord, visual pathway) during guiding clinical treatment. Therefore, we continued to use these OAR constraints. Follow-up to 2017 showed that the total rNPC group in our center had a survival rate of 82.2% without local progression [4], proving that our criteria can balance tumor control and OAR toxicity. However, the goal of treatment is to maximize the control of tumors maximizing organ safety. The conversion results in this study showed that OAR constraints at our center appeared too conservative since no neurotoxic side effects were reported. It is likely that the dose prescription could be increased while OAR constraints could be slightly decreased to better treat highly radio-resist tumors. All future work using the two models could be based on the conversion curve we obtained without the need for additional physical conversion studies.

The patient plan used in the study was not generated by the original Syngo treatment plan system, but rather generated in Raystation. Although the plan itself meets the clinical plan evaluation criteria, it still differs from the actual treatment plan. This may affect the target and OAR dose conversion results. Our method of converting OAR constraints from our 21-fraction treatment to the NIRS 16-fractions treatment involved converting the LEM RBE-weighted dose from 21 to 16 fractions using the LQ model. We then converted from the LEM 16-fractions dose to the MKM 16-fraction dose using the conversion curve. The conversion results still need to be validated by subsequent clinical studies.

## Conclusions

Using the isovolumetric dose method, we converted the LEM RBE-weighted doses for actual patients with rNPC into MKM RBE-weighted doses in targets, moreover, established a conversion curve extended to OAR stand.

The OAR constraints on rNPC that we experienced at our center were proven safe after converting to MKM RBE-weighted dose, referred to NIRS and considering re-irradiation. Either LEM or MKM CIRT could allow our constraints to safely treat rNPC without additional conversion studies. What’s more, the dose constraints for other critical organs or re-irradiations could be derived or verified following our formula. The LEM or MKM clinical experiences could be translated using the corresponding biophysical model. This reduces the overall cost for deriving the OAR constraints for the whole CIRT community.

## Data Availability

The datasets used and analyzed during the current study are available from the corresponding author on reasonable request.

## Abbreviations

CIRT: Carbon-ion radiotherapy
CTV: Clinical target volume
CNAO: National Center of Oncological Hadrontherapym, Italy
cc: Cubic centimeter
GTV: Gross tumor volume
LEM: Local effect model
LEM prescription: Prescription in LEM plan
LEM RBE-weighted dose: RBE-weighted dose calculated by means of LEM
LQ: Linear-quadratic
MKM: Microdosimetric kinetic model
MKM RBE-weighted dose: RBE-weighted dose calculated by means of MKM
NIRS: National Institute of Radiobiological Sciences
OAR: Organ at risk
OAR constraints: RBE-weighted dose constraints on OARs
PTV: Planning target volume
rNPC: Recurrent nasopharyngeal carcinoma
SPHIC: Shanghai Proton and Heavy Ion Center
